# Incidence, mortality, and factors associated with primary postpartum haemorrhage following in-hospital births in northwest Ethiopia

**DOI:** 10.1371/journal.pone.0266345

**Published:** 2022-04-06

**Authors:** Bewket Tiruneh, Ensieh Fooladi, Gayle McLelland, Virginia Plummer

**Affiliations:** 1 School of Nursing and Midwifery, Monash University, Clayton, Melbourne, Australia; 2 School of Nursing, University of Gondar, Gondar, Ethiopia; 3 School of Health, Federation University, Berwick, Melbourne, Australia; The University of Mississippi Medical Center, UNITED STATES

## Abstract

**Background:**

Of the 1010 reported maternal deaths in 2018, just over 65% occurred in hospitals in Ethiopia. However, there is a lack of standardised data about the contributing factors. This study aimed to investigate the incidence, mortality, and factors associated with primary postpartum haemorrhage following in-hospital births in northwest Ethiopia.

**Methods:**

A retrospective cohort design was used; an audit of 1060 maternity care logbooks of adult women post-partum at Felege Hiwot Referral Hospital and University of Gondar Comprehensive Specialized Hospital. The data were abstracted between December 2018 and May 2019 using a systematic random sampling technique. We used the Facility Based Maternal Death Abstraction Form containing sociodemographic characteristics, women’s medical history, and partographs. Primary postpartum haemorrhage was defined as the estimated blood loss recorded by the staff greater or equal to 500 ml for vaginal births and 1000 ml for caesarean section births, or the medical doctor diagnosis and recording of the woman as having primary postpartum haemorrhage. The data analysis was undertaken using Stata version 15. Variables with P ≤ 0.10 for significance were selected to run multivariable logistic analyses. Variables that had associations with primary postpartum haemorrhage were identified based on the odds ratio, with 95% confidence interval (CI) and P-value less than 0.05.

**Results:**

The incidence of primary postpartum haemorrhage in the hospitals was 8.8% (95% CI: 7.2, 10.6). Of these, there were 7.4% (95% CI: 2.1, 13.3) maternal deaths. Eight predictor variables were found to be independently associated with primary postpartum haemorrhage, including age ≥35 years (AOR: 2.20; 95% CI: 1.08, 4.46; P = 0.03), longer than 24 hours duration of labour (AOR: 7.18; 95% CI: 2.73, 18.90; P = 0.01), vaginal or cervical lacerations (AOR: 4.95; 95% CI: 2.49, 9.86; P = 0.01), instrumental (forceps or vacuum)-assisted birth (AOR: 2.92; 95% CI: 1.25, 6.81; P = 0.01), retained placenta (AOR: 21.83; 95% CI: 6.33, 75.20; P = 0.01), antepartum haemorrhage in recent pregnancy (AOR: 6.90; 95% CI: 3.43, 13. 84; p = 0.01), women in labour referred from primary health centres (AOR: 2.48; 95% CI: 1.39, 4.42; P = 0.02), and births managed by medical interns (AOR: 2.90; 95% CI: 1.55, 5.37; P = 0.01).

**Conclusion:**

We found that while the incidence of primary postpartum haemorrhage appeared to be lower than in other studies in Africa the associated maternal mortality was higher. Although most factors associated with primary postpartum haemorrhage were consistent with those identified in the literature, two additional specific factors, were found to be prevalent among women in Ethiopia; the factors were referred women in labour from primary health facilities and births managed by medical interns. Maternal healthcare providers in these hospitals require training on the management of a birthing emergency.

## 1. Introduction

Although the introduction of effective medical treatment, such as oxytocin administration to prevent labour and birth complications has reduced maternal mortality rate (MMR) significantly worldwide, MMR is still unacceptably high, especially in low-resource countries [1]. An estimate on MMR for 2017 shows that, on average, 211 women died per 100,000 live births globally, with a 415 MMR occurring in low-resource countries [1]. Sub-Saharan African nations, including South Sudan (1150 MMR), Chad (1140 MMR), and Sierra Leone (1120 MMR), are on top of the world list of countries with the highest MMR [1]. Of 121 countries, Ethiopia was ranked 33 in 2019 by MMR rank of countries [2]. Compared with other African countries, such as Egypt (37 MMR), Namibia (195 MMR), and neighbouring Djibouti (248 MMR), Ethiopia has the highest MMR (401MMR) in 2017 [1]. Of the 1010 reported maternal deaths, the majority (71.7%; 725) occurred in healthcare organisations, with 65.1% (658) in hospitals and 6.6% (67) in health centres in 2018 [3]. Approximately 86% of maternal mortality worldwide was due to direct maternal causes, including postpartum haemorrhage, infections, pre-eclampsia or eclampsia, obstructed labour and ectopic pregnancy [4]. Postpartum haemorrhage alone causes 25% of women’s deaths [5], with the majority occurring within 24 hours following birth [6], highlighting that primary postpartum haemorrhage (PPPH) remains the principal cause of maternal mortality in the world.

PPPH is defined as the loss of blood from the genital tract in excess of 500ml following vaginal birth or 1000ml at caesarean section within the first 24 hours following birth [7]. Severe PPPH is defined as greater than 1000mls of blood loss within the 24 hours following birth [7].

Poor uterine tone accounts for the majority of PPPH (80%), followed by genital tract trauma (13%), retained uterine products (5%), and coagulation disorders (2%) [7, 8]. There are various factors that increase a woman’s risk of experiencing a PPPH. They range from sociodemographic factors to clinical factors [7]. Sociodemographic factors include age and ethnicity (e.g. from African nations). Clinical factors include medical conditions such as hypertensive disorders of pregnancy, anaemia, multiple pregnancy or current clinical practices (e.g. instrumental birth and induction of labour) [7]. The discharge of women following in-hospital births before completing 24 hours of postpartum care linked to a shortage of resources, such as beds, mainly in a low-resource country, such as Ethiopia may increase the risk of PPPH’s morbidity and mortality. It is because in some women, the occurrence of PPPH is unpredictable [9].

In response to the high MMR across the country, the Ethiopian government introduced successive national reproductive health strategies, targeting the improvement of quality and equity of health services including training additional healthcare staff and encouraging safe birth utilisation [10]. The Federal Ministry of Health of Ethiopia also introduced free ambulance services specifically for the transportation of women in labour to primary health centres. When required, women with perinatal complications, these ambulances are used to transportation to referral hospitals with higher care facilities [11]. Despite these efforts, the deaths of women following in-hospital births continue to be a major public health problem for the country [3].

There are multiple reasons for the higher maternal death rate in Ethiopia [12], which can be explained by the “Three Delays” model [13]. This model was first introduced by Thaddeus and Maine in 1994 to understand the social determinants of maternal mortality [13] and the model is useful to explain the complex issues surrounding maternal death rates in low resource countries. Delay one is a delay in deciding to seek care, which encompasses women’s socioeconomic characteristics, such as income. Delay two is the delay in reaching the health facility, due to accessibility of healthcare facilities. Delay three is the delay in receiving quality care once at the healthcare facility due to organisational related problems, for example, shortages of resources, including experienced healthcare providers [13].

In Nigeria, evidence showed that the incidence of PPPH was 34% [14]. This reflects the level of care provided to women in a low-resource country, where many women do not have access to skilled attendants during childbirth [15]. The reported incidence of PPPH in other African countries ranges from 10% in Rwanda [16] to 1.6% in Zimbabwe [17]. In Ethiopia, studies have reported that the magnitude of PPPH ranges from 12.9% [18] to 16.6% [19]. However, the data from these studies may include secondary postpartum haemorrhage, which occurs 24 hours after birth. Moreover, the collection of perinatal and MMR data in tertiary referral hospitals of Ethiopia is not standardised, making access to reliable data regarding factors contributing to PPPH challenging to ascertain. The aim of this study was to investigate the incidence, mortality, and factors associated with PPPH following in-hospital births in Amhara National Regional State, northwest Ethiopia.

## 2. Materials and methods

### 2.1 Study design

This retrospective cohort study design was part of a mixed-methods study, designed to investigate the incidence, mortality and factors associated with PPPH following in-hospital births in northwest of Ethiopia. The quantitative results of the investigation are presented in accordance with the Guidelines for Strengthening the Reporting of Observational Studies in Epidemiology [20].

### 2.2 Study setting

The study setting was two tertiary referral hospitals in Amhara National Regional State namely Felege Hiwot Referral Hospital and the University of Gondar Comprehensive Specialized Hospital located in Gonar and Bahirdar Cities, Ethiopia. These two tertiary referral hospitals jointly provide care to more than 12 million people in the region. They provide maternal healthcare, with approximately 16,000 births in 2019 [21, 22]. In all levels of public healthcare settings, the density of medical doctors and midwives per 10,000 population was 0.29 and 0.30, respectively in this region [23].

### 2.3 Study population

This study used discharged women’s maternity care logbooks as a study population. The records of women aged 18 years and above who had given birth in the hospital between December 2018 and May 2019 were included in this study. Maternity care logbooks of women aged below 18 years or those with indicating they had given birth in another health service and had transferred to the study setting or those which had been transferred to another department were excluded from the study.

### 2.4 Sampling technique

The sampling technique was *systematic* random sampling. The maternity care logbook of the first woman to be included in the sample was selected by lottery method from the first five logbooks, numbered one to five and number two was selected. Starting with maternity care logbook number two, every fifth logbook was included in the study, until the proposed sample size (1060) was reached.

### 2.5 Sample size

A single population proportion formula was used to determine a sample size to estimate the proportion or percentage of an outcome of interest in a population from data obtained from sample [24], and the following assumptions were made: The proportion of PPPH 6% [25], a confidence level of 95%, a margin of error 1.5%. The lowest margin of error (1.5%) considered is by taking into consideration the variations of the proportion of PPPH. Therefore, by considering 10% expected incomplete records, the final minimum sample size was 1060.

### 2.6 Data collection and study instrument

Data were collected between December 2018 and May 2019 using a three part pre-existing tool from the National Technical Guidance for Maternal and Perinatal Death Surveillance and Response Team of Ethiopia [26]. The first part of the tool contains sociodemographic characteristics of women, while the second component records the obstetric characteristics of the woman, with causes contributing to maternal death. The third component of the tool records the management of birth at the hospitals. To support the quality, completeness, and integrity of the extracted data, the first author reviewed the logbook audit form daily and missing data added if retrieved.

The women’s maternity care logbook is their comprehensive medical record in the hospital. The data include socio-demographic characteristics, medical and surgical history, complications in the recent pregnancy, during labour, birth, and postpartum. This logbook also contains a summary of hospital management of birth and investigative results, and information about hospital discharge (time, date, and caregiver’s name). Referral notes (if any) will also be integrated with this logbook, along with full details. Referral time and date, address, relevant medical or surgical history, findings from physical examination and tests, suspected diagnosis, any treatment given at lower healthcare organisations, and the reason for referring the women to higher healthcare facilities were included in the referral note.

### 2.7 Outcome measurement

The diagnosis of the woman’s PPPH was informed by one of two methods. The first was by the estimated blood loss recorded by the staff, greater or equal to 500 ml for vaginal births and 1000 ml for caesarean section births. The second was the medical staff diagnosis and recording of the woman as having PPPH.

### 2.8 Statistical analysis

Frequency distributions and percentages were calculated to describe sociodemographic characteristics. Multivariate statistical means were used to explore associations between PPPH and independent variables, and a P value less than or equal to 0.1 was further analysed in the multivariate logistic regression, fitted to control the possible effect of confounders. Odds ratios and 95% CI were used as measures of association. A P-value <0.05 in the multivariable model were accepted as statistically significant. Stata version 15 was used to analyse the data (StataCorp. 2017. Stata Statistical Software: Release 15. College Station, TX: StataCorp LLC.).

### 2.9 Ethics approval

Prior to commencing data collection, ethical approval to conduct the study was provided by Monash University (14113) and the University of Gondar (O/V/P/RCS/05/497). The human research ethics committees granted a waiver of consent for using retrospective data as the women were already discharged from the hospital, and contacting them was not possible.

## 3. Results

### 3.1 Sociodemographic characteristics of the study participants

In this study, a total of 1060 women’s maternity care logbooks were assessed. The age of the participants ranged from 18 to 45 years, with a mean (SD) of 26.6 (5.1) years. Eight in ten (80%) women were under the age of 31, reflecting the general population for women in Ethiopia [27]. A higher number of the participants were aged between 20–25 (40.8%, 432) and 26–30 years (36.3%, 385). The group with lowest number of participants, was also the youngest in age at 18 and 20 years (5.6%, 59). Seven in ten of the participants were living in urban areas (70.4%, 746), while nearly one in three (29.6%, 314) lived in rural areas. Most of the participants (99.3%, 1053) were married and gave birth after admission to the hospital (97.4%, 1032). Over three quarters (82.5%, 874) of women were discharged from the birth unit within 24 hours of childbirth. More than half of women (50.2%, 532) reported gave birth for the first time. The remaining women (49.8%, 528) were multigravidas. Of these, the majority had between one and four children (45.1%, 478). The remaining had over four children (4.7%, 50). Other sociodemographic characteristics of study participants are shown in Table 1.

**Table 1:** Sociodemographic characteristics of study participants, Amhara National, Regional State Referral Hospitals, Ethiopia, 2018 (n = 1060).

Variables		Frequency (%)
Age (years)	18–20	59 (5.6)
	20–25	432 (40.8)
	26–30	385 (36.3)
	31–45	184 (17.4)
Places of residences	Urban	746 (70.4)
	Rural	314 (29.6)
Marital status	Married	1053 (99.3)
	Unmarried and divorced	7 (0.7)
	None	532 (50.2)
Number of living children	1–4	478 (45.1)
	>4	50 (4.7)
Antenatal care	Yes	999 (94.2)
	No	61 (5.8)
Duration of hospitalisation	<24 hours	874 (82.5)
	≥24 hours	186 (17.5)
Time of birth	While waiting for care	28 (2.6)
	Following the start of care	1032 (97.4)

### 3.2 Incidence and mortality of PPPH

Overall, out of the 1060 casebooks assessed, 94 women experienced a PPPH. Therefore, the incidence of PPPH was 8.9% (95% CI: 7.2, 10.6). Of 94 women, 53.2% (n = 50) of the women lived in urban areas. Over two thirds (n = 67; 71.3%) of the women who experienced PPPH were between the age of 20 and 30 years. For these women, PPPH was caused by uterine atony (n = 69; 73.4%), retained placenta (n = 13; 13.8%), and trauma to birth canal (n = 12; 12.8%). Sixty-six women lost an estimated or measured amount of blood of 500–1000 ml, while 20 women had over 1000 ml of blood loss, which is severe PPPH. The data was incomplete for eight women. After examining the management approaches used for these women; nine in 10 women were managed by uterotonic agents and resuscitation (n = 84; 89.4%), while the remaining were managed by lifesaving surgical procedure, including laparotomy and/or hysterectomy (n = 10; 10.6%).

Among the 94 women who experienced PPPH, death was the outcome for seven (7.4%, 95% CI: 2.1, 13.3). Using the ‘Three Delays Model’ model [13] to analyse the contributing factors for these deaths revealed that all causes of maternal deaths arose from one of these three delays. The majority (n = 4, 57%) of women’s deaths appeared to be caused by the delay in reaching the right level of care. One third of the women’s (n = 2, 29%) deaths were attributed to delay in providing quality medical treatment. One maternal death was because of the delay in seeking medical care.

### 3.3 Factors associated with PPPH

The univariate and multivariate logistic regression analysis of factors correlated with PPPH following in-hospital births are summarised in Table 2. Of 16 predictor variables that showed association with PPPH during univariate logistic regression analysis, only eight predictor variables were found independently associated with PPPH at P < 0.05 in the multivariate logistic regression analysis. These variables were age of women at birth ≥35 years (AOR: 2.20; 95% CI: 1.08, 4.46; P = 0.03), duration of labour longer than 24 hours (AOR: 7.18; 95% CI: 2.73, 18.90; P = 0.01), vaginal and/or cervical lacerations (AOR: 4.95; 95% CI: 2.49, 9.86; P = 0.01), instrumental (forceps or vacuum)-assisted birth (AOR: 2.92; 95% CI: 1.25, 6.81; P = 0.01), retained placenta (AOR: 21.83; 95% CI: 6.33, 75.20; P = 0.01), antepartum haemorrhage in recent pregnancy (AOR: 6.90; 95% CI: 3.43, 13. 84; p = 0.01), women in labour referred from primary health centres (AOR: 2.48; 95% CI: 1.39, 4.42; P = 0.01), and births managed by medical interns (AOR: 2.90; 95% CI: 1.55, 5.37; P = 0.01).

**Table 2:** Univariate and multivariate logistic regression analysis of factors associated with primary postpartum haemorrhage following in-hospital births, 2018 (n = 1060).

Variables		PPPH		Odds ratio (OR) with 95% CI		P-value
		Yes	No	COR	AOR	
Age at birth	<35	77	880	1	1	
	≥35	17	86	2.26 (1.28, 3.40) *	2.20 (1.08, 4.46) **	0.03
Residence	Urban	50	696	1	1	
	Rural	44	270	2.27 (1.48, 3.48) *	1.31 (0.75, 2.28)	0.96
Parity	Nulliparous	32	435	1	1	
	Multiparous	62	531	1.59 (1.02, 2.48) *	1.67 (0.96, 2.88)	0.07
Attended antenatal care	Yes	82	917	1	1	
	No	12	49	2.74 (1.40, 5.35) *	2.03 (0.88, 4.70)	0.10
History of eclampsia/pre-eclampsia	Yes	15	88	1.89 (1.05, 3.43) *	1.21 (0.60, 2.48)	0.61
	No	79	878	1	1	
History of stillbirth or neonatal loss	Yes	13	64	2.26 (1.19, 4.28) *	0.66 (0.30, 1.46)	0.30
	No	81	902	1	1	
History of spontaneous abortion	Yes	16	70	2.63 (1.46, 4.74) *	1.60 (0.71, 3.45)	0.26
	No	78	896	1	1	
PROM	No	82	776	1	1	
	Yes	12	190	0.60 (0.32, 1.12)	0.47 (0.22, 1.01)	0.53
The onset of labour	Spontaneous	68	793	1	1	
	Induced	26	173	1.75 (1.08, 2.83) *	1.66 (0.90, 3.07)	0.11
The duration of labour	≤24 hours	83	947	1	1	
	>24 hours	11	19	6.60 (3.04, 14.35) *	7.18 (2.73, 18.90) **	0.01
Vaginal or cervical lacerations	Yes	20	69	3.51 (2.02, 6.10) *	4.95 (2.49, 9.86) **	0.01
	No	74	897	1	1	
Retained placenta	Yes	12	6	23.42 (8.57, 64.00) *	21.83 (6.33, 75.20) **	0.01
	No	82	960	1	1	
Transportation used	Ambulance	20	68	0.28 (0.16, 0.49) *	1.03 (0.48, 2.18)	0.94
	Public	74	898	1	1	
APH in recent pregnancy	Yes	19	41	5.72 (3.16, 10.34) *	6.90 (3.43, 13. 84) **	0.01
	No	75	925	1	1	
Type of referral	Health facility	72	468	3.48 (2.13, 5.71) *	2.48 (1.39, 4.42) **	0.02
	Self-referral	22	498	1	1	
Mode of birth	Spontaneous vaginal	66	775	1	1	
	Instrumental	13	38	4.02 (2.04, 7.91) *	2.92 (1.25, 6.81) **	0.01
	caesarean section	15	153	1.15 (0.64, 2.07)	0.76 (0.36, 1.60)	0.47
Birth managed by	Medical intern	75	480	4.00 (2.38, 6.72) *	2.90 (1.55, 5.37) **	0.01
	Staff	19	486	1	1	

Note.

* = statistically significant at P < 0.05, 1 = reference, APH = antepartum haemorrhage, AOR = adjusted odds ratio, COR = crude odds ratio.

## 4. Discussion

This study aimed to determine incidence, mortality and factors associated with PPPH following in-hospital births, in the tertiary referral hospitals of Amhara National Regional State, northwest Ethiopia. The results from the current study show that the reported incidence of PPPH following in-hospital births in the participant hospitals was 8.9%. This finding is similar to a study conducted in Uganda (9%) [28]. However, it is higher than the results of studies from other countries with similar resourcing, including Chad (1.5%) [29], Zimbabwe (1.6%) [17], Nigeria (3.4%) [30], and Egypt (3.7%) [31]. Although the differences are difficult to explain, variation in blood loss estimation [32] may underpin the difference in the results between the current study and previous studies in Zimbabwe [17] Nigeria [30], Chad [29], and Egypt [31].

Conversely, compared to other countries in Africa and Asia, including Ghana (20.3%) [33], India (14.8%) [34] and Pakistan (14.5%) [35], the incidence of PPPH in this study is low. Several reasons may have contributed to the comparatively low-reported incidence of PPPH in this study. First, most women were discharged from the birth unit before completing 24 hours of care after childbirth due to bed shortage, and blood loss for the remaining 24 hours postpartum is unrecorded, so their final blood loss outcomes were unknown. Second, depending upon where they lived, it was highly possible that women experiencing PPPH after discharge attended another hospital on their return journey rather than returning to the birth hospital. With no data linkage system between health services in Ethiopia [36], records are likely to be incomplete especially for those discharged under 24 hours. Considering this, the reported incidence of PPPH is potentially lower than the incidence found. This highlights the importance of the development of a unified identification system that collects birth data across all sources to provide a reliable and consistent database about birth statistics including PPPH. Furthermore, it is important that maternity healthcare staff have the resources to ensure standardised data is collected. An example would be the provision of scales or calibrated bags so actual blood loss during and after birth can be measured rather than estimated by visualisation estimation [37]. With such tools objective data can be obtained and response to PPPH can occur without delay. Healthcare workers should also be supported to educate all childbearing women to identify all the signs indicating that they may be experiencing an abnormal blood loss and how to manage this event including obtaining immediate medical assistance. Ultimately, this should aid to reduce the risk of delay in the seeking, reaching, and receiving care.

The provision of safe postpartum care for women in their local area by supporting healthcare organisations with the required resources could be the better solution. Potentially, those women could continue receiving in-hospital support following birth locally for at least 24 hours. However, this would mean more resourcing for additional maternity beds. In the meantime, those women who are discharged in less than 24 hours postpartum from the referral hospital, especially those from remote areas, could be accommodated close to the hospital [38]. Introduction of maternity reforms with the implications on resources balanced against the needs of the women requires further research.

In this study, most of the factors associated with the occurrence of PPPH among women in northwest Ethiopia were similar to those found in several other studies undertaken in low-resource settings [18, 19, 25, 39, 40]. In line with studies conducted in Ethiopia [18, 19, 25], this study shows an association between the age of women (≥35 years), instrumental-assisted birth as well as longer than 24 hours of labour and PPPH. Similarly, consistent with a study conducted in Ethiopia [19] and Afghanistan [39], the result of the current study demonstrated that antepartum haemorrhage in recent pregnancy and vaginal or cervical laceration was a risk factor for the incidence of PPPH. This study also found retained placenta to be a risk factor for the incidence of PPPH, which was similar to a study conducted in Rwanda [40]. This study identified two critical factors aligned to the “Three Delays” model [13], for maternal morbidity in northwest Ethiopia.

The first factor identified is “delay in reaching the health facility”. Women in labour who were referred to tertiary referral hospitals by primary health centres were more likely to experience PPPH than self-referred women. This finding suggests that delay in reaching care was a contributing factor for the morbidity of women in northwest Ethiopia. In this region, women’s transportation to tertiary referral hospitals is affected by a shortage of transport service providers, together with long distances [41]. This can create delays for the arrival at hospital for definitive management. Whilst, transportation issues are not unique to Ethiopia, the introduction of the ambulance service for women in labour is relatively new for the country. A review of the effectiveness and utilisation of the ambulance service is essential. There may be a need to provide skilled care during transportation either having the maternity care provided by a skilled healthcare professional or upskilling the ambulance attendants. Further investigation is required to determine what would be suitable for this region.

The second factor identified is “delay in receiving quality care”. The results of this study show that women whose births were managed by medical interns were more likely to experience PPPH than those whose births were managed by qualified staff (medical doctors or midwives). Due to a lack of skilled healthcare personnel in Ethiopia, it is not unusual for medical interns to provide direct healthcare services without supervision in various clinical areas of the hospitals, including birthing units [42]. As this has direct clinical consequences, there is an urgent need to address this practice. Whilst the optimal solution would be by increasing the availability of skilled midwifery and obstetric staff would be the best solution, this is unlikely in the short term. In the meantime, other educational and resourcing strategies should be introduced to ensure junior staff are supported. Strategies need to include supportive supervision by qualified clinicians and frequent evidence-based education with the multidisciplinary team training for birthing emergencies to aid in improving outcomes [9, 43, 44].

According to the findings of the current study, 7.4% of 94 women who had PPPH died. The mortality of women from PPPH in this study was low compared to the results from other studies conducted in low-resource countries, including Uganda (27%) [45], Tanzania (27%) [46] and Rwanda (31%) [16]. The disparity in results between the current study and Tanzania’s study [46] could be owing to seasonal variation of birth in sub-Saharan Africa [47]. With seasonal variation of childbirth, the risk of women to develop PPPH varied. In turn, this directly contributed to the disparity of the mortality from PPPH. These may be the reasons for the variation of results between the current study in Ethiopia and Tanzania’s study [46].

On the other hand, the methodological differences could explain the disparity between this study’s results and those conducted in Rwanda [16] and Uganda [45]. The researchers in Rwanda and Uganda collected data prospectively. The prospective study design allowed the researchers to obtain complete data by asking the medical staff and study participants or attendants for any missing data. In contrast, the researcher in the current study collected the data retrospectively. In a retrospective research method, issues with incomplete data cannot be addressed using this approach. As a result, the reported mortality of women from PPPH in the current study appeared low compared to the studies in Rwanda [16] and Uganda [45]. A prospective study in northwest Ethiopia is warranted.

Conversely, the mortality of women from PPPH in this study was high compared to the findings from other countries in Africa, including Nigeria (1.5%) [48] and (1.6%) [49], and Senegal and Mali (5.4%) [50]. The variation between the current study and studies from other African countries may reflect the socioeconomic status of these countries. Unlike Ethiopia, Nigeria and Senegal [51] are classed as middle-income countries where some women have access to a better equipped health system. The medical resources in Ethiopian tertiary referral hospitals provide less access to medications, equipment and blood for transfusions [42]. The lack of resources in Ethiopia also hinders the introduction and implementation of the required education to support maternity healthcare providers for obstetric emergencies [9]. Ultimately the lack of resources hinders the ability to fund the necessary changes required to provide safe and quality maternity healthcare services in tertiary referral hospitals in northwest Ethiopia which can explain the differences found with the studies from Nigeria and Senegal. These findings highlight the importance of further investigations to examine the best way to provide safe and quality maternity healthcare services. These will include but are not limited to studies investigating the distribution of healthcare supplies in this region; the current clinical management of birthing complications, and the implementation of the recommended practices for prevention and safe treatment of PPPH. Further investigations of these and other factors are needed to inform the introduction of strategies to reduce the morbidity and mortality of PPPH in the future.

## 5. Strengths and limitations

This study has the following strengths. First, it used a surveillance tool to collect data. Second, the sampling technique was systematic random sampling; hence, the risk of selection bias is minimised. Finally, as this study used a larger sample size, the findings could be indicative of other referral hospitals in Amhara National Regional State of Ethiopia. However, this study has limitations. This retrospective cohort study used routinely collected hospital data. Thus, the results were highly dependent on the quality of data acquired during a woman’s admission to a hospital for labour and birth. The discharge of most women within 24 hours of birth meant that women who developed PPPH after discharge may have sought care at another healthcare facility, hence, their ongoing care documentation was not linked. Thus, the overall incidence of PPPH and associated deaths reported may be underestimated in this study.

## 6. Conclusions and recommendations

The findings in this study reflected that the incidence of PPPH appeared to be lower than in other studies in Africa; however, the associated maternal death was high. While most factors associated with PPPH were like those identified in past research, two specific factors, referred women in labour from primary health facilities and births managed by medical interns, were found to be prevalent among women in Ethiopia. Considering these findings, the following strategies are recommended for prevention and reduction of PPPH. and thereby reduction in MMR. Women in Ethiopia should have access to in-hospital and/or community midwifery support following birth for at least 24 hours. Direct clinical care should be provided or adequately supervised by qualified practitioners who must also provide regular workplace education on the management of birthing emergencies in all levels of health services providing these services. Maternal healthcare providers, including primary health centre staff will require additional training on identifying risk of PPPH and transferring the woman as early as possible. Finally, the midwifery services of these hospitals should have access to emergency equipment, intravenous fluids, blood, and medications for resuscitative measures necessary during the management of PPPH. Further prospective study would provide a greater understanding about the reasons childbearing women die in tertiary referral hospitals in northwest Ethiopia.

## Supporting information

S1 Checklist (DOC)

S1 Table Univariate and multivariate logistic regression analysis of factors associated with PPPH following in-hospital births in northwest Ethiopia (n = 1060). (DOCX)

S1 Data (DTA)
