# Supplementary material for: Incidence, mortality, and factors associated with primary postpartum haemorrhage following in-hospital births in northwest Ethiopia
Source: PLoS One. 2022 Apr 6;17(4):e0266345. doi: 10.1371/journal.pone.0266345 (PMC8986012; doi:10.1371/journal.pone.0266345)
Supplement: S1 Checklist — (DOC) [file pone.0266345.s001.doc]

STROBE report.

|  | Item No | Recommendation | Page | Relevant text from manuscript |
| --- | --- | --- | --- | --- |
| **Title and abstract** | 1 | (*a*) Indicate the study’s design with a commonly used term in the title or the abstract | 1 | The type of study is indicated in the  abstract |
|  |  | (*b*) Provide in the abstract an informative and balanced summary of what was done and what was found | 1 | The abstract contains a description of the investigation. |
| Introduction | | | |  |
| Background/rationale | 2 | Explain the scientific background and rationale for the investigation being reported | 2-6 | Background and rationale are reported in the article. |
| Objectives | 3 | State specific objectives, including any prespecified hypotheses | 6 | The objective of the study is indicated in the introduction. |
| Methods | | |  |  |
| Study design | 4 | Present key elements of study design early in the paper | 7 | Study design is reported |
| Setting | 5 | Describe the setting, locations, and relevant dates, including periods of recruitment, exposure, follow-up, and data collection | 7 | Study setting is reported |
|  |  |  | 9 | Periods of recruitment and data collection are reported |
| Participants | 6 | (*a*) *Cohort study*—Give the eligibility criteria, and the sources and methods of selection of participants. Describe methods of follow-up  *Case-control study*—Give the eligibility criteria, and the sources and methods of case ascertainment and control selection. Give the rationale for the choice of cases and controls  *Cross-sectional study*—Give the eligibility criteria, and the sources and methods of selection of participants | 7 | A retrospective cohort study; eligibility criteria and methods of selection are detailed in the methods section. |
|  |  | (*b*) *Cohort study*—For matched studies, give matching criteria and number of exposed and  unexposed  *Case-control study*—For matched studies, give matching criteria and the number of controls per case |  |  |
| Variables | 7 | Clearly define all outcomes, exposures, predictors, potential confounders, and effect modifiers. Give diagnostic criteria, if applicable | 9 | The outcome variable is defined, and measurement described |
|  |  |  | 16 | Possible confounders are identified and presented using table. |
| Data sources/ measurement | 8* | For each variable of interest, give sources of data and details of methods of assessment (measurement). Describe comparability of assessment methods if there is more than one group | 9 | The content of the tool and the sources of data are described. |
| Bias | 9 | Describe any efforts to address potential sources of bias | 9 | To support integrity of the extracted data, the logbook audit form reviewed daily and missing data added if retrieved. |
| Study size | 10 | Explain how the study size was arrived at | 9 | A single population proportion formula was used to determine the required sample size. |
| Quantitative variables | 11 | Explain how quantitative variables were handled in the analyses. If applicable, describe which groupings were chosen and why | 16 | During the analysis the quantitative variables were handled as “yes” for cases or “no” for non-cases |
| Statistical methods | 12 | (*a*) Describe all statistical methods, including those used to control for confounding | 9 | Multivariate logistic regression analysis described. Odds ratios and 95% confidence intervals were used as measures of association. P-values less than <0.05 in the multivariable model were accepted as statistically significant. |
|  |  | (*b*) Describe any methods used to examine subgroups and interactions |  | Not applicable |
|  |  | (*c*) Explain how missing data were addressed |  | Missing data were removed in the analysis. |
|  |  | (*d*) *Cohort study*—If applicable, explain how loss to follow-up was addressed  *Case-control study*—If applicable, explain how matching of cases and controls was addressed  *Cross-sectional study*—If applicable, describe analytical methods taking account of sampling strategy |  |  |
|  |  | (*e*) Describe any sensitivity analyses | 8 and 9 | The criteria for inclusion in the study described, the definition of the outcome variable provided, confounding variables were identified, missing data removed, and samples were selected at regular intervals using the discharge sequences of women from the hospital as a sampling frame |

Continued on next page

| Results | | |  |  |
| --- | --- | --- | --- | --- |
| Participants | 13* | (a) Report numbers of individuals at each stage of study—eg numbers potentially eligible, examined for eligibility, confirmed eligible, included in the study, completing follow-up, and analysed | 10 | The total number of logbooks assessed is reported |
|  |  | (b) Give reasons for non-participation at each stage |  | Not applicable |
|  |  | (c) Consider use of a flow diagram |  |  |
| Descriptive data | 14* | (a) Give characteristics of study participants (eg demographic, clinical, social) and information on exposures and potential confounders | 10 | Socio-demographic characteristics of the study participants are reported and analysed |
|  |  | (b) Indicate number of participants with missing data for each variable of interest |  | None |
|  |  | (c) *Cohort study*—Summarise follow-up time (eg, average and total amount) |  |  |
| Outcome data | 15* | *Cohort study*—Report numbers of outcome events or summary measures over time | 10 | The numbers of the outcome are reported |
|  |  | *Case-control study—*Report numbers in each exposure category, or summary measures of exposure |  |  |
|  |  | *Cross-sectional study—*Report numbers of outcome events or summary measures |  |  |
| Main results | 16 | (*a*) Give unadjusted estimates and, if applicable, confounder-adjusted estimates and their precision (eg, 95% confidence interval). Make clear which confounders were adjusted for and why they were included | 16 | Multivariate logistic regression analysis described. Crude Odds Ratio, Adjusted Odds Ratio and 95% confidence intervals were used as measures of association. |
|  |  | (*b*) Report category boundaries when continuous variables were categorized |  | The age of the women was categorised |
|  |  | (*c*) If relevant, consider translating estimates of relative risk into absolute risk for a meaningful time period |  |  |
| Other analyses | 17 | Report other analyses done—eg analyses of subgroups and interactions, and sensitivity analyses |  | Nil. |
| Discussion | | |  |  |
| Key results | 18 | Summarise key results with reference to study objectives | 19 | The key results of the study are summarised in relation to the objective of the study. |
| Limitations | 19 | Discuss limitations of the study, taking into account sources of potential bias or imprecision. Discuss both direction and magnitude of any potential bias | 24 | The limitation of the study reported |
| Interpretation | 20 | Give a cautious overall interpretation of results considering objectives, limitations, multiplicity of analyses, results from similar studies, and other relevant evidence | 19-24 | The discussion of results was with consideration of the limitation of the data source, sample size and definition of the outcome variables. |
| Generalisability | 21 | Discuss the generalisability (external validity) of the study results | 25 | The generalisability of the result discussed |
| Other information | | |  |  |
| Funding | 22 | Give the source of funding and the role of the funders for the present study and, if applicable, for the original study on which the present article is based | 26 | This study was unfunded |

*Give information separately for cases and controls in case-control studies and, if applicable, for exposed and unexposed groups in cohort and cross-sectional studies.

**Note:** An Explanation and Elaboration article discusses each checklist item and gives methodological background and published examples of transparent reporting. The STROBE checklist is best used in conjunction with this article (freely available on the Web sites of PLoS Medicine at http://www.plosmedicine.org/, Annals of Internal Medicine at http://www.annals.org/, and Epidemiology at http://www.epidem.com/). Information on the STROBE Initiative is available at www.strobe-statement.org.
