# Supplementary material for: Incidence, mortality, and factors associated with primary postpartum haemorrhage following in-hospital births in northwest Ethiopia
Source: PLoS One. 2022 Apr 6;17(4):e0266345. doi: 10.1371/journal.pone.0266345 (PMC8986012; doi:10.1371/journal.pone.0266345)
Supplement: S1 Table — (DOCX) [file pone.0266345.s002.docx]

| Variables | | PPPH | | Odds ratio (OR) with 95% CI | | P-value |
| --- | --- | --- | --- | --- | --- | --- |
|  |  | Yes | No | COR | AOR |  |
| Age at birth | <35 | 77 | 880 | 1 | 1 |  |
|  | ≥35 | 17 | 86 | 2.26 (1.28, 3.40) * | 2.20 (1.08, 4.46) ** | 0.03 |
| Residence | Urban | 50 | 696 | 1 | 1 |  |
|  | Rural | 44 | 270 | 2.27 (1.48, 3.48) * |  |  |
| Parity | Nulliparous | 32 | 435 | 1 |  |  |
|  | Multiparous | 62 | 531 | 1.59 (1.02, 2.48) * |  |  |
| Attended antenatal care | Yes | 82 | 917 | 1 |  |  |
|  | No | 12 | 49 | 2.74 (1.40, 5.35) * |  |  |
| History of eclampsia/pre-eclampsia | Yes | 15 | 88 | 1.89 (1.05, 3.43) * |  |  |
|  | No | 79 | 878 | 1 |  |  |
| History of stillbirth or neonatal loss | Yes | 13 | 64 | 2.26 (1.19, 4.28) * |  |  |
|  | No | 81 | 902 | 1 |  |  |
| History of spontaneous abortion | Yes | 16 | 70 | 2.63 (1.46, 4.74) * |  |  |
|  | No | 78 | 896 | 1 |  |  |
| PROM | No | 82 | 776 | 1 |  |  |
|  | Yes | 12 | 190 | 0.60 (0.32, 1.12) |  |  |
| The onset of labour | Spontaneous | 68 | 793 | 1 |  |  |
|  | Induced | 26 | 173 | 1.75 (1.08, 2.83) * |  |  |
| The duration of labour | ≤24 hours | 83 | 947 | 1 |  |  |
|  | >24 hours | 11 | 19 | 6.60 (3.04, 14.35) * | 7.18 (2.73, 18.90) ** | 0.01 |
| Vaginal or cervical lacerations | Yes | 20 | 69 | 3.51 (2.02, 6.10) * | 4.95 (2.49, 9.86) ** | 0.01 |
|  | No | 74 | 897 | 1 | 1 |  |
| Retained placenta | Yes | 12 | 6 | 23.42 (8.57, 64.00) * | 21.83 (6.33, 75.20) ** | 0.01 |
|  | No | 82 | 960 | 1 | 1 |  |
| Transportation used | Ambulance | 20 | 68 | 0.28 (0.16, 0.49) * |  |  |
|  | Public | 74 | 898 | 1 |  |  |
| APH in recent pregnancy | Yes | 19 | 41 | 5.72 (3.16, 10.34) * | 6.90 (3.43, 13. 84) ** | 0.01 |
|  | No | 75 | 925 | 1 | 1 |  |
| Type of referral | Health facility | 72 | 468 | 3.48 (2.13, 5.71) * | 2.48 (1.39, 4.42) ** | 0.02 |
|  | Self-referral | 22 | 498 | 1 | 1 |  |
| Mode of birth | Spontaneous vaginal | 66 | 775 | 1 | 1 |  |
|  | Instrumental | 13 | 38 | 4.02 (2.04, 7.91) * | 2.92 (1.25, 6.81) ** | 0.01 |
|  | caesarean section | 15 | 153 | 1.15 (0.64, 2.07) | 0.76 (0.36, 1.60) | 0.47 |
| Birth managed by | Medical intern | 75 | 480 | 4.00 (2.38, 6.72) * | 2.90 (1.55, 5.37) ** | 0.01 |
|  | Staff | 19 | 486 | 1 | 1 |  |

1= reference category
